# The Association of Gut Microbiota with Nonalcoholic Steatohepatitis in Thais

**DOI:** 10.1155/2018/9340316

**Published:** 2018-01-16

**Authors:** Abhasnee Sobhonslidsuk, Suwannee Chanprasertyothin, Tanjitti Pongrujikorn, Piyaporn Kaewduang, Kwannapa Promson, Supanna Petraksa, Boonsong Ongphiphadhanakul

**Affiliations:** ^1^Division of Gastroenterology and Hepatology, Department of Medicine, Faculty of Medicine, Ramathibodi Hospital, Mahidol University, Bangkok, Thailand; ^2^Office of Research Academic and Innovation, Faculty of Medicine, Ramathibodi Hospital, Mahidol University, Bangkok, Thailand; ^3^Division of Endocrinology, Department of Medicine, Faculty of Medicine, Ramathibodi Hospital, Mahidol University, Bangkok, Thailand

## Abstract

**Objectives:**

Nonalcoholic steatohepatitis (NASH) can progress to advanced fibrosis; the link between intestinal bacterial overgrowth and NASH has been proposed. Gut microbiota may promote inflammation and provoke disease progression. We evaluated gut microbiota pattern in NASH and its influencing factors.

**Methods:**

A case-controlled study with sixteen NASH and eight control subjects was done. We performed DNA extraction from stool samples and bacterial 16S rRNA sequencing using MiSeq™. The sequences were clustered into operational taxonomic units using Quantitative Insights Into Microbial Ecology software. We calculated relative abundances, determined alpha diversity, obtained beta diversity by principal coordinate analysis, and conducted the partial least-squares regression model.

**Results:**

The relative abundance of Bacteroidetes tended to be higher in NASH group. The Bacteroidetes/Firmicutes (B/F) ratio was significantly elevated in NASH patients. The pattern of gut microbiota in NASH was clearly separated from that of control subjects. Factors influencing the separation of NASH from control subjects were age, diabetes, body mass index, Bacteroidetes phylum, metformin, Actinobacteria, Verrucomicrobia, Thermotogae, and* Caldithrix* and Bacteroidetes/Firmicutes ratio.

**Conclusions:**

Bacteroidetes phylum (*Bacteroides* and* Prevotella* genus) is abundant in NASH subjects, who exhibited an elevated B/F ratio. NASH patients showed a specific pattern of gut microbiota independent of diabetes or metformin use.

## 1. Introduction

Nonalcoholic fatty liver disease (NAFLD), a condition in which the liver is composed of over 5% fat, is a significant cause of chronic liver disease worldwide [1]. Based on a recent meta-analysis study, the global prevalence of NAFLD is estimated to be approximately 25.2% [1]. The prevalence of NAFLD is increased in some groups of patients, such as individuals with type 2 diabetes, obesity, hypertension, dyslipidemia, or polycystic ovarian syndrome [1–4]. The spectrum of NAFLD ranges from simple steatosis to nonalcoholic steatohepatitis (NASH), which can progress to cirrhosis and hepatocellular carcinoma [4]. A recent study revealed that 41% of NASH patients had fibrosis progression and that 9% of the cohort developed advanced fibrosis and cirrhosis [1]. Once cirrhosis occurs in NASH patients, the annual cumulative risk of hepatocellular carcinoma can reach 2.6% [1, 4]. Nonalcoholic fatty liver disease, especially NASH, has been associated with liver-specific and overall mortality [1].

Fat deposition in the liver is regarded as the first hit in the “two-hit hypothesis” of the pathogenesis of NASH [5]. Insulin resistance, oxidative stress, inflammatory cytokine, and endotoxin release from gut microbiota can perpetuate inflammatory pathway processes, resulting in fibrosis progression towards the advanced stages of NASH [5–7]. The “multi-hit” model, which suggests that many “hit” factors can act simultaneously and result in the development and progression of NASH, was subsequently proposed in 2010 [6]. An association between NAFLD and gut microbiota has been known from the link between intestinal bacterial overgrowth and NASH [8]. Gut microbiota may influence the development of liver inflammation and NASH through Toll-like receptors and short-chain fatty acids and the production of gut hormones such as glucagon-like peptide 1 [6, 9]. A distinct pattern of gut microbiota in NAFLD patients compared with health controls has been reported [9, 10]. Zhu et al. used sequencing techniques to reveal an abundance of alcohol-producing gut bacteria with an increase in ethanol concentration in blood circulation resulting in the progression of NASH [9]. In this study, NASH patients exhibited an increased abundance of the Bacteroidetes and Proteobacteria phyla, which was mainly explained by the increased abundance of the Enterobacteriaceae family [9]. Most of the Enterobacteriaceae sequences belonged to ethanol-producing bacteria,* Escherichia* [9]. Boursier et al. identified that* Bacteroides* was independently related to NASH and that* Ruminococcus* was associated with advanced fibrosis [11]. However, Mouzaki et al. found a lower percentage of Bacteroidetes in NAFLD patients compared with normal controls using the quantitative polymerase chain reaction (qPCR) technique to study gut microbiota [10]. Due to these conflicting results, we aimed to investigate the pattern of gut microbiota in NASH versus healthy controls and to identify the independent factor(s) associated with the distinctive pattern of gut microbiota in patients with NASH.

## 2. Materials and Methods

### 2.1. Study Design

We carried out a case-controlled study at the liver unit and research center of Ramathibodi Hospital (Bangkok, Thailand) between 1 October 2015 and 30 September 2016. The study was approved by the Committee on Human Rights related to Research Involving Human Subjects, Faculty of Medicine, Ramathibodi Hospital (ID 02-52-39), and carried out according to the 1975 Declaration of Helsinki. Informed consent was acquired from the study subjects prior to their enrollment.

### 2.2. Subjects

Patients who were diagnosed with NASH based on a liver biopsy were invited to participate in the study. We carried out a histological assessment of NASH according to Brunt's and Kleiner's criteria [12]. Exclusion criteria included regular alcohol consumption, hepatitis B or hepatitis C viral infection, or liver disorders from other causes. Healthy control patients did not have diabetes, dyslipidemia, or hypertension. We collected demographic data from each patient. Body mass index (BMI) was calculated from weight (in kilograms) divided by height squared (in meters) [13].

The study subjects arrived at the hospital in the morning after a 10–12-hour overnight fast and underwent a 24-hour dietary assessment that consisted of an interview, transient elastography (TE), and a blood draw. Stool samples were collected and transported to the hospital on that same day.

### 2.3. Transient Elastography

Patients with NASH and healthy control subjects underwent TE (Fibroscan®, Echosen, Paris) to measure liver stiffness (LS) and controlled attenuation parameter (CAP), which reflected the degree of liver fibrosis and liver fat, respectively [14]. The cutoff levels of LS for hepatic fibrosis stage ≥ F2, ≥F3, and ≥F4 were 7.0, 8.7, and 10.3 kPa [14]. Control subjects were required to have CAP level less than 239 dB/m prior to enrollment in the study [15].

### 2.4. Laboratory Testing

Blood samples were taken for biochemical testing that included a complete blood count, a liver function test, glucose, glycated hemoglobin (HbA1C), and a lipid panel.

### 2.5. Stool Collection

Stools were collected in a plastic container with a tightly closing lid and stored in an insulated bag with cooling elements in the morning before being immediately transported to the hospital. At the laboratory unit, the stools were stored at −80°C for further analysis [10, 16, 17].

### 2.6. DNA Extraction

Bacterial DNA from stool was extracted using a QIAamp® Fast DNA stool mini kit (Qiagen, Duesseldorf, Germany) according to manufacturer's instruction [18]: the sample was mixed with InhibitEX buffer. Then, the supernatant was mixed with proteinase K and AL buffer. After that, ethanol was added to QIAamp spin column and centrifuged. Then, column was washed with AW1 and AW2 buffer and DNA was eluted with ATE buffer. DNA concentration was measured using Qubit® 2.0 Fluorometer (Life Technologies, Oregon, USA) and stored −20°C for further analysis.

### 2.7. 16S Ribosomal RNA Sequencing and Microbiome Analysis

The 16S rRNA was sequenced using MiSeq (Illumina Inc., California, USA). The 16S metagenomic sequencing library preparation followed the manufacturer manual. We amplified DNA for the 16S rRNA region V3-V4 using the following primer set:

  16S Amplicon PCR Forward Primer
    5′-TCGTCGGCAGCGTCAGATGTGTATAAGAGACAGCCTACGGGNGGCWGCAG-3′
  16S Amplicon PCR Reverse Primer
    5′-GTCTCGTGGGCTCGGAGATGTGTATAAGAGACAGGACTACHVGGGTATCTAATCC-3′

After the first PCR clean-up step, the samples were amplified with dual indices. We used an Illumina sequencing adapter with the Nextera XT Index Kit. The Amplicon Library was pooled, normalized, combined with PhiX Control, and sequenced.

We analyzed the taxonomic profile using 16S Metagenomics (Illumina Inc.) and Quantitative Insights Into Microbial Ecology (QIIME) (Denver, Colorado, USA) software (v 1.9.1) on a Basespace application. The relative abundance of the phylum and genus of each sample was evaluated. The sequences were clustered into taxonomic groups referred to as operational taxonomic units (OTUs) at a 97% similarity threshold based on a comparison with Greengenes data [19, 20]. Phylogenetic trees were created, and we examined the bacterial richness and diversity within samples or alpha diversities (Chao1, observed-species indices, and Shannon) and between samples or beta diversity. We calculated the ratio of Bacteroidetes to Firmicutes (B/F) [21].

### 2.8. Statistical Analysis

We compared categorical and continuous variables between groups using chi-squared, nonparametric, and Student's *t*-tests, as appropriate. A *P* value less than 0.05 was viewed as indicating statistical significance. Statistical analysis was carried out using SPSS version 16.0 (SPSS Inc., Illinois, USA). The data were analyzed using principal coordinate analysis (PCoA), based on the unweighted and weighted UniFrac distance metrics. We used the partial least-squares (PLS) regression model with R and the plsValSel package. A variable importance in projection (VIP) greater than 1 was considered to make a significant contribution to the prediction model [22–24]. A VIP score is a measure of a variable's importance in the PLS model. It is calculated as a weighted sum of the squared correlations between the PLS components and the original variable [23].

## 3. Results

### 3.1. Characteristics and Clinical Data of NASH and Normal Control Subjects

Sixteen NASH and 8 normal control subjects were recruited. A comparison of the data from the NASH and control subjects is listed in Table 1. Females dominated in both groups, and older patients were more common in the NASH group. Metabolic parameters such as diabetes, dyslipidemia, hypertension, and an increased body weight and waist circumference were more common in the NASH group. As expected, all of the subjects in the NASH group shared the typical features of metabolic syndrome. Both groups reported similar daily energy expenditures and dietary intake. NASH group had significantly higher AST and ALT levels than the control group. The mean (range) of CAP and LS levels in NASH and control subjects was 302.7 (226–389) and 185.5 (105–255) dB/m and 10.6 (4–22) and 4.7 (3–6) kPa. Ten (62.5%), 9 (56.2%), and 7 (43.8%) of NASH patients had fibrosis stage ≥ F2, ≥F3, and ≥F4, respectively.

### 3.2. Bioinformatic Data

**Figure 1:**
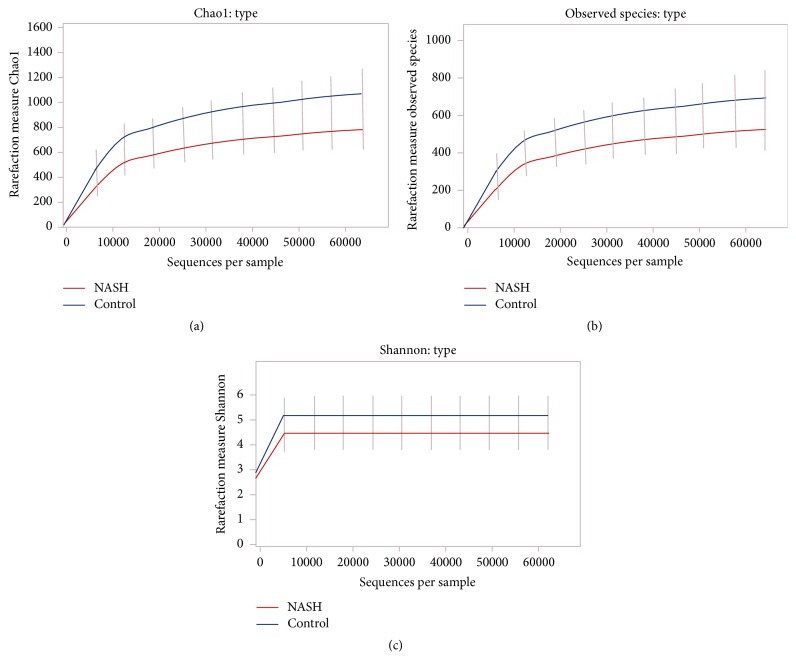
Chao1, observed species, and Shannon between NASH and control subjects. (a) Chao1, (b) observed species, and (c) Shannon.

**Figure 2:**
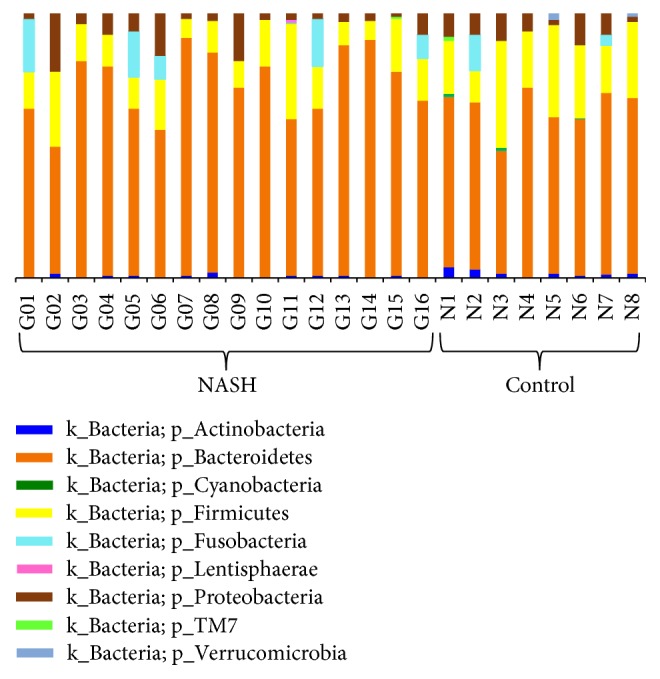
Relative abundance of phyla-level gut microbiota between NASH and control subjects.

**Figure 3:**
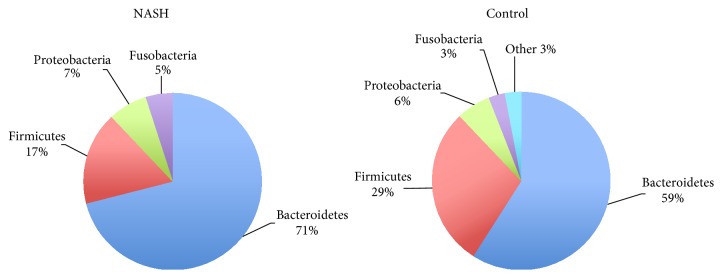
Distribution of phyla-level gut microbiota between NASH and control subjects.

**Figure 4:**
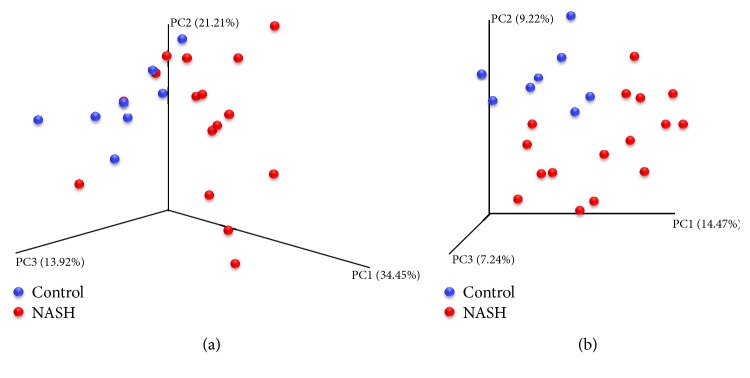
Pattern of gut microbiota in NASH versus control subjects using principal coordinate analysis (PCoA) according to the (a) unweighted and (b) weighted UniFrac distance metrics.

There were 3,629,536 high-quality reads in the total samples from 16 NASH and 8 healthy control subjects. The median number of sequences in each sample was 154,725 (range: 69,458–226,612). Among the total number of 3,606,583 bacteria from the 16 NASH and 8 control subjects, the average number of bacteria in each individual was 150,274 ± 43,500. Alpha diversity at the genus and species levels tended to be lower in patients with NASH, but the differences between both groups did not attain statistical significance (Figure 1). The relative abundance of gut microbiota at the phylum level of each subject is shown in Figure 2. Bacteroidetes was the most abundant gut microbiota in both groups, followed by Firmicutes. The phylum-level distribution of gut microbiota in the NASH patients and healthy controls is shown in Figure 3. The NASH group exhibited a larger number of Bacteroidetes phyla than the normal group (*P* = 0.002). The number of Bacteroidetes ratioed to the total number of bacteria tended to be higher in the NASH group (*P* = 0.098). The number of Firmicutes phyla tended to be lower in the NASH group, which might be explained by a smaller number of* Ruminococcus* genera. As expected, the B/F ratio was significantly elevated in the NASH group compared with the control group (5.2 ± 2.7 versus 2.3 ± 0.9, *P* = 0.005). In addition, a significantly lower number of Actinobacteria phyla were noted in the NASH group. We calculated the relative abundance of gut microbiota of each subject at the genus level. The number of* Bacteroides* and* Prevotella* genera considered to be representative of the Bacteroidetes phylum [25] was elevated in the NASH group but not significantly so, although the number of* Prevotella* was distinctively high in the NASH group (Table 2). The* Phascolarctobacterium* genus was significantly more prevalent in the NASH group. The pattern of gut microbiota of NASH subjects was clearly distinct from that in the control subjects according to PCoA using the unweighted and weighted UniFrac distance metrics (Figures 4(a) and 4(b)).

### 3.3. Identification of Key Factors Responsible for the Differentiation between NASH and Control Subjects

**Figure 5:**
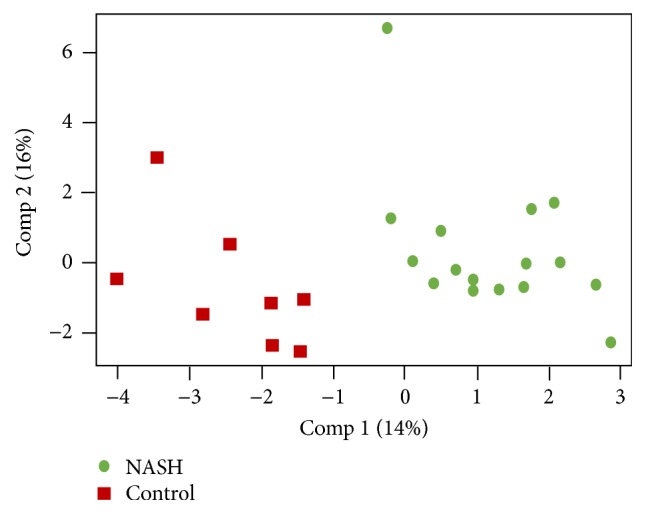
Partial least-squares (PLS) regression model reveals the clear separation of gut microbiota between NASH and control subjects.

We considered a PLS regression model with the gut microbiota at the phylum level and age, gender, BMI, diabetes, and the use of metformin as independent variables. In a score plot of components 1 and 2, we found that subjects with NASH were well separated from the control subjects based on the distribution of gut microbiota (Figure 5). To determine the importance of variables in the PLS model, we assessed the VIP score. We included the first two components of the PLS model in this analysis. Our results are listed in Table 3. Factors that were found to be important variables influencing the ability to classify subjects based on NASH status included age, diabetes, BMI, Bacteroidetes, the use of metformin, Actinobacteria, Verrucomicrobia, Thermotogae, and* Caldithrix*. Since Bacteroidetes and Firmicutes have been reported to be important factors associated with NAFLD [9–11], we performed additional analyses using the B/F ratio. The important variables influencing the ability to classify subjects based on NASH status included age, BMI, and diabetes. When subjects on metformin and diabetes were excluded, we found that the B/F ratio was an important factor for the separation of the gut microbiota pattern between NASH and normal subjects in addition to age and BMI.

### 3.4. The Significance of Disease Severity on Gut Dysbiosis in NASH Subjects

The distributions of Bacteroidetes, Firmicutes, and Proteobacteria and the percentage of Bacteroidetes in NASH subjects with advanced fibrosis (LS ≥ 8.7 kPa) and individuals with nonadvanced fibrosis (LS < 8.7 kPa) did not differ. The influence of the degree of LS on gut dysbiosis in NASH patients could not be demonstrated in this study.

## 4. Discussion

The NASH subjects in this study exhibited an increased prevalence of diabetes (68.8%), hypertension (62.5%), and dyslipidemia (56.3%), all of which are typical features of metabolic syndrome. Gut dysbiosis has generally been accepted to be a pathogenetic factor of NAFLD and NASH [8–11, 22, 26]. Studies involving gut microbiome alterations in patients with NASH, however, are rare. We found that the Bacteroidetes and Proteobacteria phyla were more prevalent in NASH patients, despite a lack of statistical significance for Proteobacteria. This result is similar to that of previous reports [9, 11]. Zhu et al. found that Bacteroidetes and Prevotella increased together with ethanol-producing bacteria (i.e., Proteobacteria/Enterobacteria/*Escherichia*) [9]. In this study, the number of* Escherichia* species tended to increase in the NASH group, consistent with the findings of a previous report [22]. However, studies of gut microbiota in NAFLD individuals have not yielded consistent results [10]. An inverse association between the presence of NAFLD and the percentage of Bacteroidetes was reported by Mouzaki et al. [10]. An increased abundance of the* Bacteroides* genus and/or gut dysbiosis has been reported in NASH patients with more advanced fibrosis [11]. The majority of NASH patients in this study had LS > 7 kPa, which corresponded to fibrosis stage ≥ 2 or significant fibrosis, which may explain the abundance of Bacteroidetes phylum. Furthermore, different sequencing methods for assessing gut microbiota may yield opposite trends [10, 19]. Given the decline in Firmicutes species in the NASH group, we expected that the B/F ratio would be significantly elevated in this group. However, an increase in the B/F ratio may be confounded by the underlying metabolic syndrome, which has also been associated with an increase in such a ratio [27, 28]. Moreover, the use of glucose-lowering agents, metformin in particular, has been implicated as an important contributing factor to the inconsistencies found among studies of gut microbiota and metabolic syndrome [29–31]. In this study, we demonstrated the influence of gut microbiota in classifying subjects according to the presence or absence of NASH. Furthermore, we have shown that gut microbiota was still associated with the presence of NASH, even when subjects with diabetes or receiving metformin were excluded. In particular, the influence of the Bacteroidetes phylum persisted in each analysis that we performed. Taken altogether, our findings suggest that the patterns of gut microbiota are linked to NASH, independent of diabetes or metformin use. Whether such a relationship is truly causal necessitates further investigation.

Besides disease and host status, external factors like diet might affect gut microbiota. Based on the 24-hour dietary intake recall assessment, food intake in terms of total calories and the pattern of food intake between NASH patients and control subjects did not differ. However, underreporting is an important limitation of using a 24-hour dietary intake assessment [32]. A study in chimps, however, demonstrated that external factors play a much more predominant role than heritable factors [33].

Unlike Boursier et al. [11], we were unable to find the association of the severity of NASH and gut dysbiosis in our study. The small number of patients in each of our cohort groups might explain this phenomenon along with the different method to assess the severity of liver disease. Our inability to immediately freeze the stool samples, which is regarded as the gold standard of stool collection for gut microbiome assays, may be an additional limitation of this study [19]. However, thus far, there has been no evidence that techniques of stool storage contribute to changes in gut microbiota composition [34, 35].

## 5. Conclusions

Bacteroidetes phylum (*Bacteroides* and* Prevotella* genus) is abundant in NASH subjects, who exhibited an elevated B/F ratio. The dominant presence of Bacteroidetes in NASH patients is reasonably distinct from healthy subjects, independent of metabolic factors and metformin use.

## Figures and Tables

**Table 1:** Demographic and laboratory data of NASH and control subjects.

	NASH	Control	*P*
Number	16	8	
Age^*∗*^ (yr)	59.8 ± 9.6	43.4 ± 6.8	<0.001
Female (*n*, %)	13 (81.3)	8 (100)	0.277
Diabetes (*n*, %)	11 (68.8)	0	0.002
Dyslipidemia (*n*, %)	9 (56.3)	0	0.009
Hypertension (*n*, %)	10 (62.5)	0	0.004
BMI^*∗*^ (kg/m^2^)	27.7 ± 4.8	21.3 ± 1.2	0.001
WC^*∗*^ (cm)	97 ± 13.1	74 ± 7.2	<0.001
LS^*∗*^ (kPa)	10.9 ± 5.4	4.7 ± 1.5	<0.001
CAP^*∗*^ (dB/m)	302.7 ± 48.5	185.5 ± 39.0	<0.001
Energy intake^*∗*^ (kcal/day)	1258.1 ± 312.5	1,606.3 ± 476.0	0.089
Carbohydrate^*∗*^ (g/day)	190.7 ± 58.2	199.3 ± 60.5	0.743
Total fat^*∗*^ (g/day)	33.7 ± 10.7	55.6 ± 26.5	0.054
Fiber^*∗*^ (g/day)	10.7 ± 7.9	12.5 ± 7.8	0.609
AST^*∗*^ (IU/L)	49.7 ± 11.9	24.4 ± 11.6	<0.001
ALT^*∗*^ (IU/L)	59 ± 30	17 ± 6	0.004

^*∗*^Mean ± SD; NASH, nonalcoholic steatohepatitis; BMI, body mass index; WC, waist circumference; LS, liver stiffness; CAP, controlled attenuation parameter; AST, aspartate aminotransferase; ALT, alanine aminotransferase.

**Table 2:** Distribution of bacteria types at genus level between NASH and control subjects.

	NASH	Control	*P*
*Bacteroides*	75,273 ± 8,836	60,447 ± 8,676	0.246
*Prevotella*	26,407 ± 9,860	1,564 ± 1,170	0.408
*Fusobacterium*	6,213 ± 3,002	3,131 ± 7,709	0.456
*Phascolarctobacterium*	5,056 ± 969	2,214 ± 372	0.037
*Blautia*	3,291 ± 609	5,510 ± 964	0.074
*Faecalibacterium*	2,610 ± 891	6,778 ± 2,130	0.103
*Sutterella*	3,867 ± 778	3,796 ± 1,120	0.960
*Flavobacterium*	3,853 ± 885	3,384 ± 753	0.690
*Parabacteroides*	3,876 ± 674	3,278 ± 578	0.508
*Oscillospira*	3,634 ± 1,250	2,440 ± 324	0.368
*Ruminococcus*	2,201 ± 376	3,596 ± 696	0.071
*Roseburia*	993 ± 256	4,140 ± 2,484	0.245
*Escherichia*	2634 ± 1252	323 ± 137	0.086
*Paraprevotella*	2269 ± 823	603 ± 342	0.077

Data are expressed as mean ± standard error of mean (SEM) except if stated otherwise. NASH, nonalcoholic steatohepatitis.

**Table 3:** The variable importance in projection (VIP) score for the partial least-squares regression model.

Variable	VIP score
Age	2.10
Diabetes	2.02
BMI	1.82
Bacteroidetes	1.71
Use of metformin	1.55
Actinobacteria	1.34
Verrucomicrobia	1.28
Thermotogae	1.27
*Caldithrix*	1.12

## References

[1] Younossi Z. M., Koenig A. B., Abdelatif D., Fazel Y., Henry L., Wymer M. (2016). Global epidemiology of nonalcoholic fatty liver disease—meta-analytic assessment of prevalence, incidence, and outcomes. *Hepatology*.

[2] Sayiner M., Koenig A., Henry L., Younossi Z. M. (2016). Epidemiology of Nonalcoholic Fatty Liver Disease and Nonalcoholic Steatohepatitis in the United States and the Rest of the World. *Clinics in Liver Disease*.

[3] Sobhonslidsuk A., Pulsombat A., Kaewdoung P., Petraksa S. (2015). Non-alcoholic Fatty Liver Disease (NAFLD) and significant hepatic fibrosis defined by non-invasive assessment in patients with type 2 diabetes. *Asian Pacific Journal of Cancer Prevention*.

[4] Younossi Z., Henry L. (2016). Contribution of alcoholic and nonalcoholic fatty liver disease to the burden of liver-related morbidity and mortality. *Gastroenterology*.

[5] Rowell R. J., Anstee Q. M. (2015). An overview of the genetics, mechanisms and management of NAFLD and ALD. *Clinical Medicine*.

[6] Yu J., Marsh S., Hu J., Feng W., Wu C. (2016). The pathogenesis of nonalcoholic fatty liver disease: interplay between diet, gut microbiota, and genetic background. *Gastroenterology Research and Practice*.

[7] Buzzetti E., Pinzani M., Tsochatzis E. A. (2016). The multiple-hit pathogenesis of non-alcoholic fatty liver disease (NAFLD). *Metabolism*.

[8] Wigg A. J., Roberts-Thomson I. C., Dymock R. B., McCarthy P. J., Grose R. H., Cummins A. G. (2001). The role of small intestinal bacterial overgrowth, intestinal permeability, endotoxaemia, and tumour necrosis factor *α* in the pathogenesis of non-alcoholic steatohepatitis. *Gut*.

[9] Zhu L., Baker S. S., Gill C. (2013). Characterization of gut microbiomes in nonalcoholic steatohepatitis (NASH) patients: a connection between endogenous alcohol and NASH. *Hepatology*.

[10] Mouzaki M., Comelli E. M., Arendt B. M. (2013). Intestinal microbiota in patients with nonalcoholic fatty liver disease. *Hepatology*.

[11] Boursier J., Mueller O., Barret M. (2016). The severity of nonalcoholic fatty liver disease is associated with gut dysbiosis and shift in the metabolic function of the gut microbiota. *Hepatology*.

[12] Yeh M. M., Brunt E. M. (2014). Pathological features of fatty liver disease. *Gastroenterology*.

[13] Keys A., Fidanza F., Karvonen M. J., Kimura N., Taylor H. L. (1972). Indices of relative weight and obesity. *Journal of Chronic Diseases*.

[14] Wong V. W.-S., Vergniol J., Wong G. L.-H. (2010). Diagnosis of fibrosis and cirrhosis using liver stiffness measurement in nonalcoholic fatty liver disease. *Hepatology*.

[15] Sasso M., Miette V., Sandrin L., Beaugrand M. (2012). The controlled attenuation parameter (CAP): A novel tool for the non-invasive evaluation of steatosis using Fibroscan*Ⓡ*. *Clinics and Research in Hepatology and Gastroenterology*.

[16] Qin J., Li R., Raes J. (2010). A human gut microbial gene catalogue established by metagenomic sequencing. *Nature*.

[17] Tong M., Jacobs J. P., McHardy I. H., Braun J. (2014). Sampling of intestinal microbiota and targeted amplification of bacterial 16S rRNA genes for microbial ecologic analysis. *Current Protocols in Immunology*.

[18] Mirsepasi H., Persson S., Struve C., Andersen L. O. B., Petersen A. M., Krogfelt K. A. (2014). Microbial diversity in fecal samples depends on DNA extraction method: EasyMag DNA extraction compared to QIAamp DNA stool mini kit extraction. *BMC Research Notes*.

[19] van Best N., Jansen P. L., Rensen S. S. (2015). The gut microbiota of nonalcoholic fatty liver disease: current methods and their interpretation. *Hepatology International*.

[20] McDonald D., Price M. N., Goodrich J. (2012). An improved Greengenes taxonomy with explicit ranks for ecological and evolutionary analyses of bacteria and archaea. *The ISME Journal*.

[21] Andoh A., Nishida A., Takahashi K. (2016). Comparison of the gut microbial community between obese and lean peoples using 16S gene sequencing in a Japanese population. *Journal of Clinical Biochemistry and Nutrition*.

[22] Wong V. W.-S., Tse C.-H., Lam T. T.-Y. (2013). Molecular characterization of the fecal microbiota in patients with nonalcoholic steatohepatitis—a longitudinal study. *PLoS ONE*.

[23] Mehmood T., Liland K. H., Snipen L., Sæbø S. (2012). A review of variable selection methods in Partial Least Squares Regression. *Chemometrics and Intelligent Laboratory Systems*.

[24] Duft R. G., Castro A., Bonfante I. L., Brunelli D. T., Chacon-Mikahil M. P., Cavaglieri C. R. (2017). Metabolomics Approach in the Investigation of Metabolic Changes in Obese Men after 24 Weeks of Combined Training. *Journal of Proteome Research*.

[25] Mariat D., Firmesse O., Levenez F. (2009). The firmicutes/bacteroidetes ratio of the human microbiota changes with age. *BMC Microbiology*.

[26] Raman M., Ahmed I., Gillevet P. M. (2013). Fecal microbiome and volatile organic compound metabolome in obese humans with nonalcoholic fatty liver disease. *Clinical Gastroenterology and Hepatology*.

[27] Damms-Machado A., Mitra S., Schollenberger A. E. (2015). Effects of surgical and dietary weight loss therapy for obesity on gut microbiota composition and nutrient absorption. *BioMed Research International*.

[28] Stadlbauer V., Leber B., Lemesch S. (2015). Lactobacillus casei Shirota supplementation does not restore gut microbiota composition and gut barrier in metabolic syndrome: A randomized pilot study. *PLoS ONE*.

[29] Forslund K., Hildebrand F., Nielsen T. (2015). Disentangling type 2 diabetes and metformin treatment signatures in the human gut microbiota. *Nature*.

[30] Zhang X., Zhao Y., Xu J. (2015). Modulation of gut microbiota by berberine and metformin during the treatment of high-fat diet-induced obesity in rats. *Scientific Reports*.

[31] Lee H., Ko G. (2014). Effect of metformin on metabolic improvement and gut microbiota. *Applied and Environmental Microbiology*.

[32] Gemming L., Jiang Y., Swinburn B., Utter J., Mhurchu C. (2014). Under-reporting remains a key limitation of self-reported dietary intake: An analysis of the 2008/09 New Zealand Adult Nutrition Survey. *European Journal of Clinical Nutrition*.

[33] Moeller A. H., Ochman H. (2013). Factors that drive variation among gut microbial communities. *Gut Microbes*.

[34] Deda O., Gika H. G., Wilson I. D., Theodoridis G. A. (2015). An overview of fecal sample preparation for global metabolic profiling. *Journal of Pharmaceutical and Biomedical Analysis*.

[35] Tedjo D. I., Jonkers D. M. A. E., Savelkoul P. H. (2015). The effect of sampling and storage on the fecal microbiota composition in healthy and diseased subjects. *PLoS ONE*.

